# Reverse Total Shoulder Arthroplasty with a Cementless and Metaphyseal Stem Fixation Is a Viable Option for the Treatment of Proximal Humeral Fractures with Calcar Involvement

**DOI:** 10.3390/jcm12041443

**Published:** 2023-02-11

**Authors:** Raffaele Garofalo, Alberto Fontanarosa, Nunzio Lassandro, Angelo De Crescenzo

**Affiliations:** Department of Orthopaedics and Traumatology, Ente Ecclesiastico Ospedale “F. Miulli”, Strada Prov. 127 Acquaviva–Santeramo Km. 4, Acquaviva delle Fonti, 70021 Bari, Italy

**Keywords:** cementless and metaphyseal fixation, calcar, cerclage, shoulder arthroplasty, proximal humeral fracture

## Abstract

Background: The purpose of the study was to evaluate the suitability of reverse total shoulder arthroplasty (RTSA) with a cementless and metaphyseal stem fixation as a treatment for complex proximal humeral fractures (PHFs) with a calcar fragment when this may be fixed with a steel wire cerclage. Clinical and radiographic outcomes were compared with the same RTSA for PHFs without a calcar fragment at a minimum of five-year follow-up. Methods: A retrospective analysis was performed on acute PHFs “with a medial calcar fragment” (group A) and “without a calcar fragment” (group B) treated with a RTSA and cementless metaphyseal stem fixation. Results: At an average follow-up of 6.7 years (5–7.8 years), no statistical difference was observed comparing group A (18 patients) to group B (50 patients) for active anterior elevation (141 ± 15° vs. 145 ± 10°, *p* = 0.67), active external rotation ER1 (49 ± 15° vs. 53 ± 13°, *p* = 0.55), and active internal rotation (5 ± 2 vs. 6 ± 2, *p* = 0.97). Similarly, a comparison of ASES score (89.2 ± 10 vs. 91.6 ± 9, *p* = 0.23) and Simple Shoulder Test score (91.1 ± 11 vs. 90.4 ± 10, *p* = 0.49) revealed no significant difference. Conclusion: RTSA with a cementless and metaphyseal stem fixation represents a safe and feasible treatment for complex PHFs with a medial calcar fragment when this may be fixed with a steel wire cerclage.

## 1. Introduction

The treatment of proximal humeral fractures (PHFs) in elderly patients continues to be debated. Most of these fractures are managed conservatively. However, surgery is required for displaced and comminuted fractures, which can result in nonunion or malunion with consequently poor functional outcomes [1]. As shown in previous studies, complex three- and four-parts proximal humeral fractures in elderly are rarely successfully managed by internal fixation with dedicated plates for risks of avascular necrosis, non-union, loss of fixation, and hardware failure [2,3,4]. These complex fractures have been traditionally treated with hemiarthroplasty (HA) with tuberosities repair around the implant [5], but the strong influence exerted by tuberosities healing coupled with the significant rate of rotator cuff pathology determined unreliable and mixed functional outcomes [6,7,8]. Thus, reverse total shoulder arthroplasty (RTSA) has gained popularity as a treatment of PHFs in elderly patients, especially among shoulder-fellowship surgeons [9,10]. Replacing a comminuted and displaced fracture and recreating a new shoulder biomechanics less reliant on tuberosities healing and rotator cuff integrity [11,12], RTSA has achieved more consistent and satisfactory results over open reduction and internal fixation (ORIF) and HA [4].

RTSA for PHFs has been traditionally performed with a cemented stem fixation, since fractured proximal humerus is perceived not to provide adequate support for metaphyseal fixation [13]. Moreover, this approach assists surgeon in restoring anatomic humeral height and version warranting a stable primary stability. However, the cemented stem fixation introduces longer surgical time, risks of thromboembolism, higher cost, and the negative influence on tuberosities healing with exothermic reaction [6]. The bone damage is related to the thickness of the cement mantle, with a 7 mm mantle producing a peak temperature of over 55°, which is higher than the known threshold of 47° for the onset of permanent bone thermal injury [14,15]. Moreover, revision surgery may be extremely complex with potential humeral osteotomy, risks of periprosthetic fracture, and severe bone loss. Hence, stem fixation in primary surgery must always be well balanced with a potential ease removal [16,17]. This issue might become predominant in the near future since RTSA is becoming the most performed surgical treatment for these patients [18].

Accordingly, cementless RTSA has been investigated with multiple reports showing consistent clinical and radiographic outcomes [19,20,21,22,23]. These results compared similarly with those of the traditional cemented techniques [13,20,24]. Despite a wide variability on stem’s design and length, most case series have relied on standard or long cementless stems often reaching a diaphyseal fixation [19,20,21,22]. Nonetheless, shorter implants may be employed without jeopardizing stem stability [23]. With new stem design and porous coating surfaces, shorter stems with a metaphyseal fixation would provide benefits such as bone stock preservation, ease removal, and increased metaphyseal stress with accordingly reduced stress-shielding phenomena such as radiolucent lines and early loosening [23].

To date, experienced opinion published on cementless stems for PHFs has mainly dealt with three- and four-parts fractures without any report analyzing complex fractures with extension into medial calcar [23]. In these relatively rare fracture pattern, a weakened metaphysis may further compromise the primary stability of implants with a metaphyseal fixation. Hence, this scenario would foster physicians to promote a diaphyseal fixation with longer stems either cemented or not. No evidence may be hitherto found on feasibility of cementless and metaphyseal humeral fixation when fractures extend into the medial calcar.

The aim of the study was therefore to evaluate the suitability of RTSA with cementless and metaphyseal stem fixation for complex PHFs with a calcar fragment when this may be fixed with a steel wire cerclage. Clinical and radiographic outcomes at a minimum of five-years follow-up were compared with those achieved by the same RTSA in patients with PHFs without a calcar fragment. The hypothesis was that no significant difference would be found between two groups, proposing that this humeral stem is likewise safe and efficient even for complex fractures with a medial calcar fracture.

## 2. Materials and Methods

A retrospective analysis was performed on acute PHFs treated with the same cementless RTSA from January 2013 to December 2017 at a single institution. All surgeries were performed by the senior author (R.G.). Inclusion criteria were as follows: (1) minimum follow-up of five years, (2) complete preoperative radiographic data as well as those immediately after surgery and at last follow-up, (3) treatment with the same RTSA with a cementless and metaphyseal stem fixation (Ascend Flex, Stryker, Portage, MI, USA). Patients were excluded if they had chronic fractures (>4 weeks from trauma), fractures with a diaphyseal extension not amenable to being treated with a cementless and metaphyseal stem fixation, and RTSA performed for fracture malunion or avascular necrosis. The patients finally selected were divided in two groups: (group A) PHFs “with a calcar fragment” in which the calcar fragment was fixed with one or two Luque steel wire cerclages; and (group B) PHFs “without a calcar fragment”. Fractures with a medial calcar involvement were defined as all bone lesions with displaced or nondisplaced fractures of the medial proximal humerus extending or not into the meta-diaphyseal region and with or without a continuity with humeral head. Each patient gave the consent to participate to the study, which previously received the Ethics Committee approval (IRB number 6745).

Each proximal humeral fracture was studied initially with anteroposterior and Neer view X-rays performed in the emergency department. A comprehensive preoperative planning was thereafter achieved with sized bilateral full-length humeral X-rays and CT-scan. Preoperative radiographic images were evaluated by two authors (A.D.C. and A.F.).

Preoperative patients’ information recorded was demographic characteristics (age, sex, and body mass index), operated side, and fracture type. Patients were followed-up at 1, 3, 6, and 12 months and then 2 and at minimum of 5 years after surgery. Clinical examination included range of motion (ROM; anterior elevation AE, external rotation with the patient’s arm at their side ER1 and with 90° of abduction ER2, and internal rotation IR), pain score (NRS), and patient-reported outcomes (Simple Shoulder Test, SST and American Shoulder, and Elbow Surgery score, ASES). Internal rotation was measured recording the vertebral level reached by the thumb and converting it in a numerical score, with point-1 representing thigh and point-20 the first thoracic vertebra. Fractures were categorized according to the Neer classification [25]. Patients were deemed lost to follow-up and then to statistical analysis if they died, underwent revision surgery within first year of follow-up, or provided only a telephonic interview as a follow-up.

### 2.1. Implant

Each fracture was managed with the same prosthesis design, which is the Ascend Flex (Stryker, Portage, MI, USA). This is a curved shaped with collarless design prosthesis and a proximal porous coating for bone ingrowth. The stem used in this study is made of titanium and is available in eight different sizes each, ranging in length from 88 to 125 mm based on diameter. The stem inclination of 132.5° coupled with an asymmetric polyethylene insert with 12.5° of inclination leads to a humeral inclination angle of 145°. The stabilization of the stem is achieved by bone ingrowth within the compacted metaphyseal cancellous bone without diaphyseal contact of the tip of the stem.

### 2.2. Surgical Technique

Patients were placed in beach chair position after general anesthesia associated with loco-regional anesthesia. All fractures were replaced with a cementless reverse Aequalis Ascend Flex prosthesis through a standard deltopectoral approach. Once the subdeltoid space was reached, the biceps tendon was identified and prepared for later tenodesis to the upper border of the pectoralis major in all cases. The greater and lesser tuberosities were identified, isolated, and secured with no 5 nonabsorbable sutures (four for infraspinatus and teres minor, one for subscapularis) placed at the bone-tendon junction and around the tuberosity. When the tuberosities were not completely isolated by the fracture, a careful isolation with osteotome was performed trying to preserve as much cancellous bone as possible. The fractured head was then removed and saved for bone grafting.

The glenoid was thereafter approached and the glenoid component placed in the standard fashion. Care was required to implant the baseplate low or at least flush with the inferior margin of the glenoid and with a neutral or inferior inclination to reduce scapular notching. Then the baseplate was always secured with four screws.

Once the glenoid component was implanted, the humeral canal was approached. At first, the calcar fragment was evaluated for its dimension, shape, and reducibility on diaphysis without undue tension and interposition of the soft tissue. Specific care was paid to perform cleaning of the fracture’s edge and avoid excessive clearance of the soft tissue from the calcar fragment in order to improve fracture healing. In the presented series, the calcar was fractured determining a single fragment large enough to be fixed and stabilized with a metal cerclage and not excessively large to jeopardize the rotational stability of a stem with a cementless and metaphyseal fixation. At this point, one or two double 1.25 mm steel wire cerclages were passed using a shuttling device through soft tissues as close as possible to the fragment, and then around the remaining metaphysis. The cerclage was provisionally slightly tightened once a compactor or a sounder was positioned to fill the metaphysis with an accurate fit. The reduction in the metaphyseal region made it possible, in addition, to take the medial calcar as a useful landmark for proper height restoration. Then, the compactors, which have the same shape of the final implant, were used starting from the smallest to test the rotational and axial stability after the full seating. Once the stem size was defined and the definitive stem was implanted, the cerclage was tightened to ensure final primary stability and then cut.

The humeral stem was always implanted with a customed retroversion. In particular, we placed the arm in neutral rotation and in this position, we prepared the humeral shaft putting the stem face to the glenosphere. After the stem and polyethylene insert placement, and before the shoulder reduction, the deep limbs of the sutures previously placed through the posterior rotator cuff were passed around the neck of the prosthesis and through the subscapularis tendon around the lesser tuberosity. The tuberosities were thereafter gently pulled out with sutures previously prepared and the shoulder was then reduced. Once positioned around the prosthesis, the tuberosities may occasionally require debulking by removing the bone segments for a better positioning around the stem. As previously described, a standardized fixation technique for tuberosity reconstruction was performed with four horizontal and two vertical cerclages [26]. In conclusion, reverse shoulder arthroplasty was tested to confirm desired motion and stability and the incision was closed.

### 2.3. Postoperative Care

All patients underwent a standard postoperative program with the shoulder immobilized with a 30° abduction sling for the first 4 weeks. Active and passive exercises for the elbow, wrist and hand started early on after surgery as well as a scapular reconditioning program. Passive shoulder ROM was initiated in week 3 allowing gradual and pain-free elevation till 90° and for the 3 weeks afterward. External and internal rotation were both discouraged for the first 4 weeks and then gradually introduced. Active ROM began in week 6, whereas strengthening was initiated in week 10.

### 2.4. Radiological Assessment and Examination

Radiographic images were evaluated to assess any evidence of tuberosity and medial calcar healing, subsidence, radiolucent lines, implant loosening, scapular notching, development of heterotopic ossification, and periprosthetic fractures. Humeral component loosening was measured using the grading system described by Sperling [27] and defined as the presence of ≥2-mm radiolucent lines in more than three zones, whereas the Nérot–Sirveaux system was applied for the inferior scapular notching classification [28]. The bone tuberosities were considered healed in a binary fashion (“healed” or “not healed” based on the osseous union of the greater tuberosity to the proximal humeral shaft). Cases of uncertain definition were always considered “not healed”.

### 2.5. Statistical Analysis

Data were reported as mean ± standard deviation or percentage for categorical variables. A paired sample t-test was used to compare repeated measures and the non-parametric Mann–Whitney two-sample statistic to test the distribution of the values between the groups. A p-value of 0.05 or less was considered statistically significant. All analyses were conducted using STATA software, version 16 (Stata-Corp LP, College Station, TX, USA).

## 3. Results

**Figure 1:**
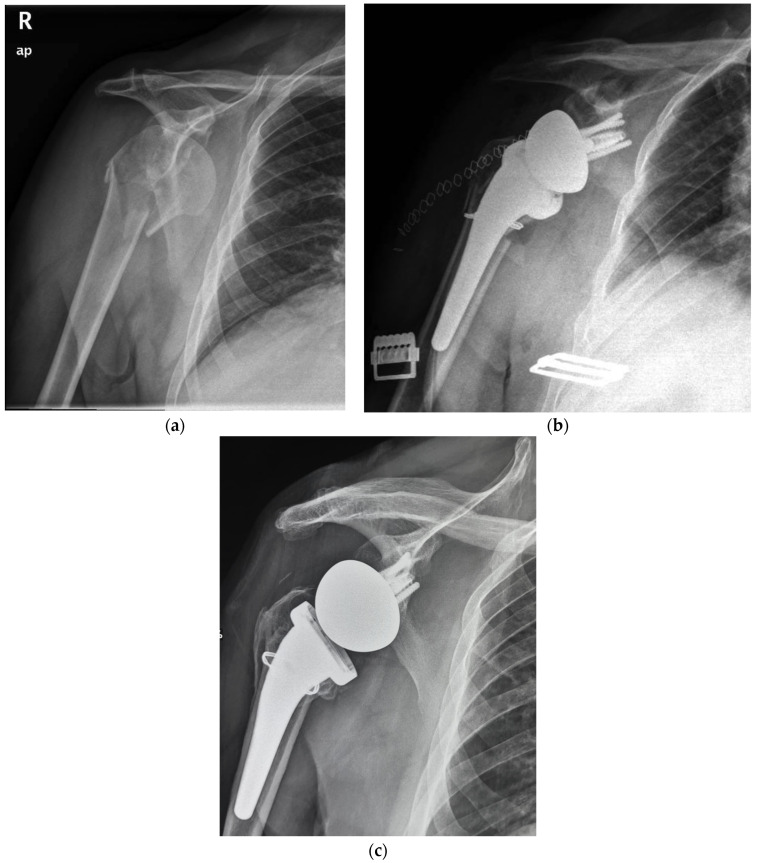
(**a**) Preoperative radiographic image of right shoulder showing acute (Neer three-part) proximal humeral fracture with calcar involvement. (**b**) Immediate postoperative film of right shoulder showing calcar fragment fixation with steel wire cerclage. (**c**) 5.7 years postoperative film of right shoulder showing stem subsidence and healing of tuberosity and medial calcar.

**Figure 2:**
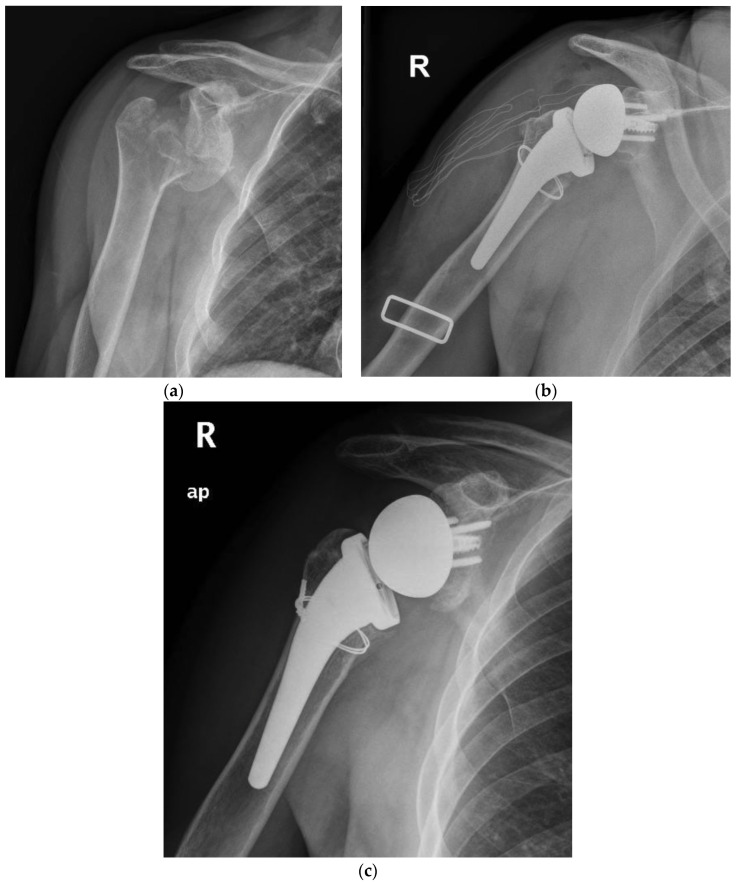
(**a**) Preoperative radiographic image of right shoulder showing acute (Neer four-part) proximal humeral fracture with calcar fragment. (**b**) Immediate postoperative film of right shoulder showing calcar fragment fixation with steel wire cerclage. (**c**) 5.5 years postoperative film of right shoulder showing healed tuberosity and medial calcar.

In the period analyzed, a comprehensive 88 cases of acute proximal humerus fractures were managed with a RTSA with a cementless and metaphyseal stem fixation in our institution. By applying the inclusion and exclusion criteria, 21 cases were finally selected for group A and 67 for group B. Two patients (9.5%) for group A and 16 (23.8%) for group B were excluded, being lost to the follow-up or for inadequate radiographic examination. Similarly, one patient for each group and one patient for group B were surgically revisited for periprosthetic fracture after a fall and a deep infection, respectively. The selection led finally to a group A of 18 patients (Figure 1 and Figure 2) and a group B of 50 patients.

The mean age was 72.3 years (range, 65–84 years) with 48 women (70%) and 20 men (30%). All patients included had an average follow-up of 6.7 years (5–7.8 years, Table 1). A statistical difference was found comparing the follow-up achieved in the two groups, with a greater follow-up for the group “without a calcar fragment” (6.9 ± 0.7 years for group A and 5.5 ± 0.5 years for group B, *p* < 0.05). According to the Neer classification, the fractures were 47 four-part (9 in group A and 38 in group B), 19 three-part (8 in group A and 11 in group B), and 2 two-part fractures (1 in group A and 1 in group B) with a split of the humeral head.

At final review, a comparison of the range of motion revealed no significant difference between the two groups (Table 2). The results were the following: active anterior elevation (141 ± 15° vs. 145 ± 10°, *p* = 0.67), active external rotation ER1 (49 ± 15° vs. 53 ± 13°, *p* = 0.55), active external rotation ER2 (35 ± 5° vs. 33 ± 5°, *p* = 0.89), and active internal rotation (5 ± 2 vs. 6 ± 2, *p* = 0.97). Similarly, a comparison of the ASES score (89.2 ± 10 vs. 91.6 ± 9, *p* = 0.23), Simple Shoulder Test score (91.1 ± 11 vs. 90.4 ± 10, *p* = 0.49), and NRS pain score (0.8 ± 2 vs. 0.5 ± 1.5, *p* = 0.58) revealed no significant difference.

At radiographic evaluation, 58 of 68 (85%) of the greater tuberosities demonstrated osseous healing (Figure 1 and Figure 2) without a significant difference observed between the two groups. A statistically significant difference was found indeed focusing on the influence of tuberosity healing on the clinical outcomes regardless of medial calcar fracture extension. The subgroup of patients with tuberosity healing achieved significant higher ASES scores and active external rotation than those without tuberosity healing (mean ASES score of 92 and 82, respectively, *p* < 0.05; mean active external rotation ER1 of 45° and 22°, respectively, *p* < 0.05).

The overall complication rate at the final follow-up was 28% (19 patients, Table 3). Postoperative complications included tuberosity non-union in ten patients, scapular notching in two patients, radiolucent lines in two patients (one in group A and one in group B), subsidence in three patients (one for group A, 5.5% and two for group B, 4%), heterotopic ossification in one patient and superficial infection in one patient. The subsidence observed (Figure 1c) was never associated with any symptoms, pain, or evident implant loosening throughout the follow-up. Bone healing of the medial calcar fragment was always observed with heterotopic ossifications in two patients.

## 4. Discussion

The goal of this study was to evaluate the safety and efficacy of a RTSA with a cementless and metaphyseal stem fixation as a treatment of complex PHFs with a medial calcar fragment when this is fixable with a steel wire cerclage. At a minimum follow-up of five years, the clinical and radiographic results were successful and similar to those achieved by the same arthroplasty design for fractures without calcar involvement. All fractures revealed a healed calcar region without any severe complication such as implant loosening.

The treatment of proximal humeral fractures in elderly patients continues to be controversial. Poor bone quality and rotator cuff pathology are the roots of the inconsistent and mixed results thus far reported with both ORIF and HA in this age bracket [2,4,6,8,29]. Conversely, RTSA has been showing more predictable and satisfying results [4,9,29,30]. As a result, complex PHFs are nowadays usually treated with RTSA in the elderly population when conservative management is not suitable [9,10].

Even though traditionally performed with cemented stems, a cementless fixation has been investigated, showing consistent and favorable clinical and radiographic results [20,21,22,23,24]. Despite a wide range of potential results, functional and radiographic outcomes are overall comparable to the traditional cemented prosthesis without any significant increase in postoperative complications, reaching a mean forward flexion of 114–157°, mean ASES scores of 48–91, and tuberosity healing rates of 60–91% [20,21,22,23,24].

To date, only two studies have directly compared cemented with uncemented stems [24]. Focusing on only one implant design, Rossi et al. recently found no significant differences in terms of active ROM, functional scores as well as tuberosities healing (56% and 60% for cemented and uncemented, respectively) at a minimum follow-up of two-years [24]. Conversely, Schoch et al. observed significantly better ASES scores and subjective satisfaction for the cemented series, but no difference in pain relief and motion recovery [20]. However, the use of four different implant systems may potentially skew the final assumptions in the latter study [20].

A recent review did not find a difference in the pain, ROM, tuberosities healing, and Constant score but a significant higher ASES score was observed in patients with the uncemented RTSA [13]. However, a greater complication rate was noted in the uncemented fixation (9.7 vs. 5.5%; *p* = 0.044), but it was not associated with any significant difference in terms of the reoperation rate (1.9% for uncemented and 1.6% for cemented fixation) [13].

The experience of cementless fixation thus far published has regarded mainly three-, four-, and rarely two-parts PHFs. No mention may be found on more severe fracture patterns, such as those with a medial calcar fragment [23]. Moreover, most of the series have employed standard or long stems with a press-fit achieved often in the diaphyseal bone. Our series supports the safety and viability of RTSA with a cementless and metaphyseal stem’s fixation for two-, three-, and four-part PHFs but as well in selected fractures with a calcar fragment that is sizable to be fixed with steel wire cerclage (Figure 1 and Figure 2). At a minimum follow-up of five years, the clinical outcomes (ASES score of 89.2, Simple Shoulder Test score 91.1 and NRS pain of 0.8) were satisfactory and supported by the comparison with a control group of patients without the calcar fragment (ASES score of 91.6, Simple Shoulder Test score 90.4 and NRS pain of 0.5). For both groups, the results are likewise comparable to those achieved by other series with either cementless or cemented stems.

In terms of complication rate, the metaphyseal and cementless fixation does not jeopardize the stem’s stability whenever the fracture’s pattern gives rise to a calcar fragment fixable with a steel wire cerclage.

As a matter of fact, a semi-constrained implant as the RTSA might result in excessive stresses on the humeral stems in elderly patients with a roomy humeral canal and a PHF. Moreover, a medial calcar disruption might further weaken metaphysis reducing primary stability especially for the uncemented humeral stems. In this scenario, the cemented stem fixation could be considered since a strong primary stability is provided with less rotational micromotion than the press-fit fixation but equivalent axial micromotion [31]. Accordingly, the press-fit stems may be potentially more prone to translation than the cemented stems, despite the evolution in the stem’s design and materials with the addition of the proximal porous coating for bone ingrowth [32]. As observed by a recent radiostereometric study, short press-fit stems are exposed to a potential mean translation of 0.6 mm more than the standard-length cemented stems at two-years follow-up, albeit not associated to any evidence of stem loosening or consequences on clinical outcomes [32].

With a complication rate similar to other reports on the press-fit stems, a 5.5% rate for either radiolucent lines and subsidence (one patient with radiolucent lines and one patient with subsidence out of 18 patients) was found in the present cohort but without any evidence of clinical consequences or stem loosening. However, no significant difference on radiolucent lines and subsidence rate was observed between the two groups analyzed (5.5 vs. 2% and 5.5 vs. 4% for group A and B, respectively, *p* = 0.85), suggesting that the safety of steel metal cerclage provides a secure and stable metaphyseal stem fixation.

In different scenarios and stem’s design or fixation, either steel or suture cerclage has been successfully tested in augmenting implant stability with humeral fractures. A recent clinical study from Eyberg et al. has shown that a suture cerclage is safe and effective to stabilize osteotomies and periprosthetic fractures in shoulder arthroplasty [33]. All cases demonstrated radiographic healing and no complications related to the cerclage fixation were observed [33]. In a case series on patients undergoing RTSA for degenerative diseases, Kriechling et al. managed intraoperative nondisplaced calcar fracture with either suture or metal cerclage achieving fracture healing without any sign of stem subsidence or loosening [34]. Moreover, as shown with a biomechanical study, the protection of the metaphyseal calcar region with steel wire cerclage results in an increased energy needed to make the fracture progress distally to the cerclage [35].

Our investigation has confirmed these findings but in the setting of acute proximal humeral fractures with a calcar fragment. A steel cerclage wire has provided a firm fragment reduction achieving an adequate metaphyseal stem fixation. In this way, a medial metaphyseal support was recreated and long cementless or cemented stems avoided. Moreover, the integrity and stability of the restored metaphyseal region may be assumed by the similar stem’s size (101.3 mm for “with a calcar fragment” group and 96.7 mm for “without a calcar fragment” group, *p* = 0.65) and accordingly the fit–fill ratio observed in the two groups. In fact, the senior author was used to place in both groups the humeral stem with the minimum size needed to achieve initial rotational and axial stability. The aim was to reach a primary metaphyseal stem stability and at same time reduce diaphyseal contact and maximize the fit–fill ratio. Thus, metaphyseal stress should be increased and stress-shielding phenomena such as radiolucent lines and early loosening reduced [36,37,38].

### Limitations

The present study has some limitations, including the retrospective design, the relatively small sample size, and the follow-up period of five years. As a matter of fact, this follow-up might be acceptable to detect the most severe complications since the greatest amount of implant translation and subsidence is observed within first year postoperatively [32]. However, a longer follow-up is certainly advocated in order to reveal radiographic complications not yet clearly evident. Then, radiographic evaluation was performed with postoperative multiplane X-rays that were not fluoroscopically controlled, and postoperative computed tomography (CT) scanning was not performed. In addition, all patients were operated on with the same implant and the same surgeon performed all procedures. Even though this represents a strength of the study for a homogeneity of the two groups crucial to draw serious conclusions, the results may not be applicable for the several other implant designs available.

## 5. Conclusions

RTSA with a cementless and metaphyseal stem fixation is a safe and feasible treatment for acute PHFs with a medial calcar fragment when this may be reduced and fixed with a steel wire cerclage. The clinical and radiographic outcomes as well as the complication rate at a minimum follow-up of five years compared similarly with those achieved in a control group of PHFs without calcar involvement.

## Figures and Tables

**Table 1:** Characteristics of study population.

Patients Demographic Characteristics		
	Data	
	With Calcar Fragment	Without Calcar Fragment
Overall, N	18	50
Age, mean ± SD, yr	69.5	70.1
Sex: male/female, n (%)	3/15	17/33
Time from injury to arthroplasty, mean, days	7.4	6.7
Operated side: left/right, n (%)	7/11 (38/62%)	24/26 (48/52%)
Follow-up, mean, years ± SD	5.5 ± 0.5	6.9 ± 0.7

Data are presented as mean ± standard deviation.

**Table 2:** Comparative analysis of postoperative clinical outcomes in proximal humeral fractures “with calcar fragment” vs. “without calcar fragment”.

Comparative Analysis of Postoperative Clinical Outcomes in Proximal Humeral Fractures “With Calcar Fragment” vs. “Without Calcar Fragment”				
		Group A	Group B	
		“With Calcar Fragment”	“Without Calcar Fragment”	*p* Value
AE		141° (15°)	145° (10°)	0.67
ER 1		49° (15°)	53° (13°)	0.55
ER 2		35° (5°)	33° (5°)	0.89
IR		5 (2)	6 (2)	0.97
NRS pain score		0.8 (2)	0.5 (1.5)	0.58
SST score		91.1 (11)	90.4 (10)	0.49
ASES shoulder score		89.2 (10)	91.6 (9)	0.23

Data are presented as mean (SD). AE, active elevation; ER, external rotation; IR, internal rotation; SST, Simple Shoulder Test; ASES, American Shoulder and Elbow Surgeons.

**Table 3:** Number and type of postoperative complications following reverse total shoulder arthroplasty with a cementless and metaphyseal stem fixation for proximal humeral fractures “with calcar fragment” vs. “without calcar fragment”.

Number and Type of Postoperative Complications Following Reverse Total Shoulder Arthroplasty with Cementless and Metaphyseal Stem’s Fixation for Proximal Humeral Fractures “With Calcar Fragment” vs. “Without Calcar Fragment”				
		Total (n = 19)	“With Calcar Fragment” (n = 5)	“Without Calcar Fragment” (n = 12)
Tuberosity healing Subsidence		10 3	3 1	7 2
Humeral radiolucency		2	1	1
Scapular notching		2	1	1
Heterotopic ossification		1	0	1
Superficial infection		1	0	1

## Data Availability

Data sharing not applicable.
